# A Strategy for Studying Environmental Engineering: Simple Hydrothermal Synthesis of Flower-Shaped Stannous Sulfide Nanomaterials for Efficient Cataluminescence Sensing of Diethyl Ether

**DOI:** 10.3390/molecules28227621

**Published:** 2023-11-16

**Authors:** Bai Sun, Jingjie Fan, Zhuo Tang, Guoji Shi, Mingjian Yi, Yun Wang, Xiangxiang Wang, Yuxian Guo, Shuguang Zhu

**Affiliations:** 1 Anhui Institute of Urban and Rural Green Development and Urban Renewal, College of Environment and Energy Engineering, Anhui Jianzhu University, Hefei 230601, China; fanf0225@163.com (J.F.); ztang022@163.com (Z.T.); shi13215555415@163.com (G.S.); mjyi@ustc.edu.cn (M.Y.); wangxiang156@126.com (X.W.); zhushuguang@ahjzu.edu.cn (S.Z.); 2 Environmental Materials and Pollution Control Laboratory, Hefei Institute of Physical Science, Chinese Academy of Sciences, Hefei 230031, China; 3 College of Mathematics and Physics, Anhui Jianzhu University, Hefei 230601, China; guoyx@ahjzu.edu.cn

**Keywords:** SnS, sensor, cataluminescence, diethyl ether detection

## Abstract

In this work, flower-like stannous sulfide (SnS) nanomaterials are synthesized using a hydrothermal method and used as sensitive materials for cataluminescence (CTL)-based detection of diethyl ether. Gas sensors based on SnS nanomaterials are prepared, and the SnS nanomaterials exhibit excellent gas-sensitive behavior towards ether. High sensitivity to ether is achieved at a relatively low operating temperature (153 °C) compared to other common sensors. The response time is 3 s and the recovery time is 8 s. The CTL intensity shows a good linear relationship (R^2^ = 0.9931) with a detection limit of 0.15 ppm and the concentration of ether in the range of 1.5–60 ppm. The proposed CTL sensor shows good selectivity towards ether. In addition, a highly stable signal is obtained with a relative standard deviation of 1.5%. This study indicates that the SnS-based sensor has excellent gas-sensitive performance and shows potential for applications in the detection of ether.

## 1. Introduction

Diethyl ether is a flammable, colorless, and transparent liquid, with a special stimulating odor, sweet taste, and volatility; the vapor and air form explosive mixtures in the presence of open flames [1,2]. When contacting air or in the right conditions, it generates potentially explosive peroxide. Diethyl ether is one of the most commonly used solvents because of its low viscosity and good water solubility, and is widely used across industries and laboratories. In the field of chemical analysis, ether is used as a solvent to extract the composition and content of substances, and then this extract is analyzed [3]; in the field of medicine, ether is mainly used for general anesthesia in recipient rats [4]. Long-term exposure to ether is harmful to the human central nervous system, liver, and other organs. Currently, ether has been listed as an occupational disease hazard factor in the field of occupational health [5,6]. Therefore, sensitive, rapid, and selective real-time monitoring of diethyl ether is very necessary [7].

There are many techniques for detecting ether, such as gas chromatography [8,9], which has good sensitivity. However, the instrument for this is complicated to operate, large in size, and cannot be used for real-time monitoring; semiconductor metal oxide sensors [10] or surface acoustic wave quartz crystal sensors [11] can also be used for detecting ether, which have good sensitivity to ether, but poor selectivity. Cataluminescence (CTL) is important in the field of chemiluminescence, which refers to the reaction of substances on the surface of catalytic materials to produce luminescence phenomena. CTL has many advantages, such as high sensitivity, fast reaction speed, high stability, no consumption of catalysts during the reaction process, and the ability to monitor a variety of gases in the environment for a long period of time. When the size of the catalyst decreases from micron to nanoscale level, the number of active sites on which the catalyst can react with analytes increase greatly. This is due to the fact that nanomaterials commonly have a high specific surface area and some surface defects, which enable the catalyst to be efficient for contacting and participating in the reaction with the reactants [12]. As a result, nanomaterials can increase the efficiency of chemiluminescence, significantly expanding the range of substances that can be monitored using chemiluminescence analysis. In addition, nanomaterials can be used to modulate their catalytic properties by changing their shape and structure. For example, modulation of catalytic activity and selectivity can be achieved by controlling the shape and size of nanomaterials [13]. This makes nanomaterials promising for a wide range of applications in catalysis. The cataluminescence method of nanomaterials has been developed for the rapid detection of volatile organic compounds in recent years due to its simple detection device, lack of a need for complex pre-treatment and enrichment processes, fast detection speed, and high sensitivity [14]. Cao [15] et al. reported that a CTL sensor utilizing a thin sheet of ZnWO_4_ could effectively detect ether; Shi [16] et al. synthesized SiO_2_/Fe_3_O_4_ microspheres to prepare an ether vapor sensor with high sensitivity and selectivity for monitoring ether; Zhang [17] et al. designed Mg–Al layered double-oxide nanomaterials to be used in CTL for a fast and highly selective ether sensor. However, the sensitivity of the materials will affect the CTL performance, and some of these sensitive materials have high costs, complex preparation processes, and relatively high operating temperatures. The development of new materials has a great impact on the development of gas sensors.

Metal sulfide nanomaterials are attractive for their unique catalytic properties, low cost, large specific surface area, excellent adsorption properties, and strong catalytic properties, making them potential alternatives to precious metals in a range of fields [18]. Stannous sulfide (SnS) is a material of wide interest with a layered orthorhombic structure of IV–VI compound semiconductors, Sn and S, which are abundant in nature [19]. Compared to 2D MoS_2_, monolayered SnS has a greater electron mobility [20]. In particular, two-dimensional sulfide-based gas sensors typically have lower operating temperatures than oxide semiconductor sensors because S atoms have smaller electronegativity and more readily absorb oxygen in the air [21,22,23,24]. Oxygen is more readily adsorbed on the surface of stannous sulfide due to its unique two-dimensional layered structure, higher free carriers, and larger specific surface area, which facilitates the CTL reaction.

In this paper, SnS nanomaterials were prepared using a hydrothermal method. We found that ether can produce strong CTL emissions on the surface of SnS nanomaterials, while there is only a weak reaction when other compounds flow through the SnS nanomaterials. Based on the CTL characteristics of ether on the surface of SnS nanomaterials, an ether gas sensor was designed. The possible mechanisms of the enhanced CTL properties of the SnS nanomaterial sensor were explored in terms of influencing factors such as temperature, concentration, and carrier gas velocity. The results showed that the SnS nanomaterials exhibited excellent CTL properties for ether detection, including superior selectivity, high sensitivity, long-term stability, fast response times, and fast recovery. This work suggests that SnS nanomaterials may be a novel CTL-sensitive material, providing a promising approach for the development of efficiently performing CTL ether sensors.

## 2. Results and Discussion

### 2.1. Material Characterization

**Figure 1:**
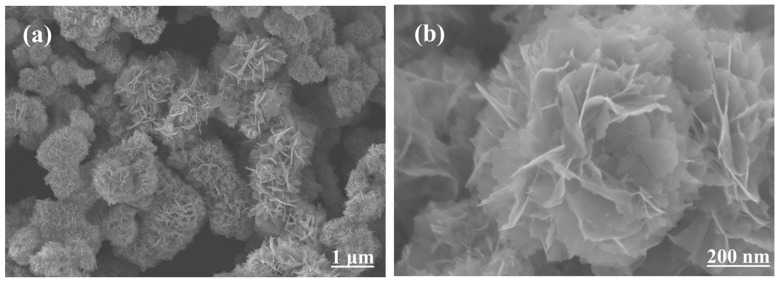
(**a**) Low-resolution SEM images of the SnS. (**b**) High-resolution SEM images of the SnS.

SEM images of the samples are shown in Figure 1. These SEM images show that the SnS has a flower-like structure with a size ranging from nanometers to micrometers. The nanoflowers are tightly clustered; the SnS nanosheets are stacked layer by layer and are closely agglomerated to form this nanoflower-like structure, which increases their surface area. From previous studies, it is known that the morphology and size of nanomaterials has an effect on CTL [25], and this special sensing property can be attributed to its special hierarchical structure and flower-like morphology. The special nanoflower-like structure [26] not only facilitates a good connection between each SnS structure, but also maintains a large retention space for gas diffusion and adsorption. In this case, most of the active centers [27] in the sensing layer consisting of SnS nanosheets can be efficiently used for the contact reaction, which significantly improves their strength. 

**Figure 2:**
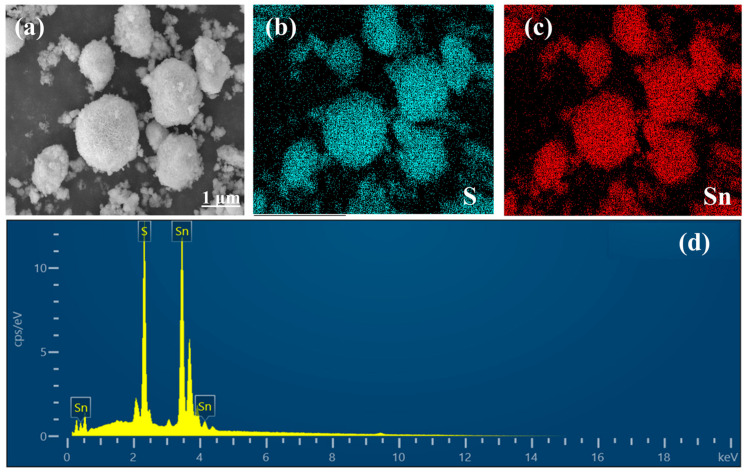
(**a**–**c**) SEM image and elemental mapping of the SnS; (**d**) EDS spectrum of the SnS.

Figure 2b,c shows the characteristic elements S and Sn of SnS. It can be observed that the elements S and Sn are uniformly distributed. Combined with the EDS energy spectrum of the sample (Figure 2d), we can clearly see its elemental composition; In Table 1, the atomic percentage between S and Sn is close to 1:1, indicating that the prepared sample is SnS, which again indicates the successful synthesis.

**Figure 3:**
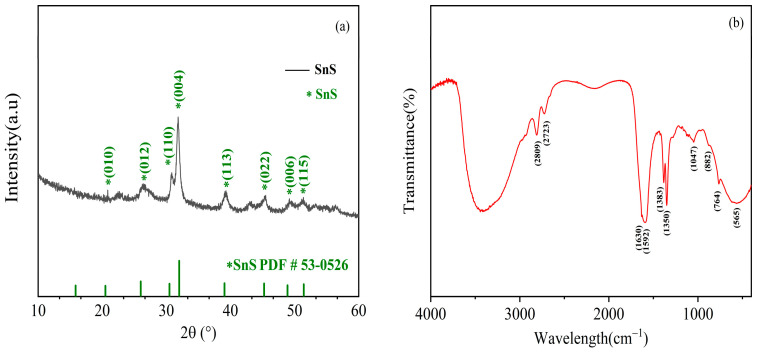
(**a**) XRD pattern and (**b**) FT-IR spectrum of flower-like SnS.

X-ray diffraction was used to examine the phase and crystallinity of the prepared SnS, as shown in Figure 3a. Seen from the XRD diffraction pattern of the nanomaterials, the diffraction peaks can be indexed to the orthorhombic crystalline SnS using standard card (PDF#53-0526). The diffraction peaks at 2θ = 20.488°, 26.003°, 30.476°, 31.995°, 39.069°,44.846°, 48.887°, and 51.432° correspond to the (010), (012), (110), (004), (113), (022), (006), and (115) crystal faces of SnS, respectively. There are no impurity peaks, indicating that the product is highly purified. Figure 3b shows the Fourier transform infrared (FTIR) spectrum of the nano-SnS in the wave number range of 4000–400 cm^−1^. The peaks at 565 cm^−1^, 764 cm^−1^, and 880 cm^−1^ are assigned to SnS stretching vibrations [28], and the peak at 1047 cm^−1^ is probably due to the partial oxidation of SnS upon exposure to air [29]. the peaks at 2809 cm^−1^ and 2723 cm^−1^ are related to the C–H stretching vibration of aldehyde. The broadband at 3500–3200 cm^−1^ is associated with the O–H stretching vibration [30].

### 2.2. Effect of Operating Temperature on CTL Intensity

**Figure 4:**
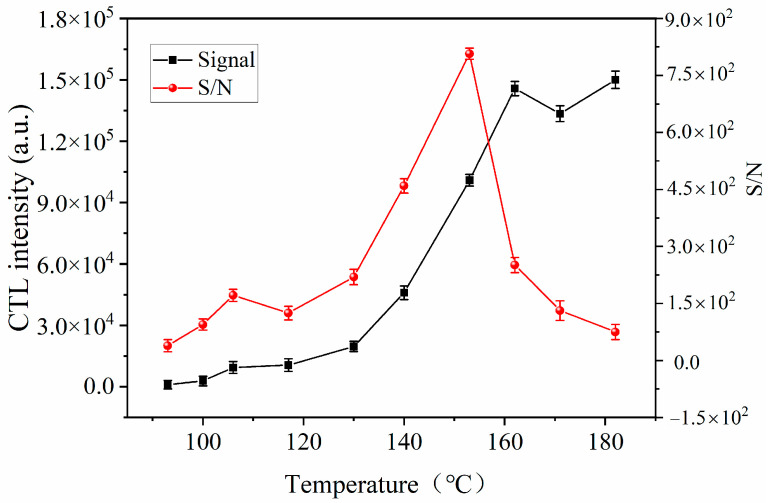
Operating temperature dependence of the CTL intensity and S/N of ether (concentration: 25 ppm; flow rate: 300 mL/min).

Temperature plays an important role in the performance of CTL. We investigated the relationship between the CTL strength of ether on the surface of SnS nanomaterials and temperature in the temperature range of 93–182 °C. The results are shown in Figure 4. Within the reaction temperature range of 93 °C to 182 °C, the CTL signal strength gradually increases depending on the increasing temperature. The signal-to-noise ratio (S/N) reaches its maximum (peak) at 153 °C. All sensors are affected by dark current. The cause of dark current is electron hole pairs generated by thermal excitation [31]. As long as its temperature is not absolute zero Kelvin, the electron hole pair inside the device is always in the dynamic equilibrium of generation, migration, and annihilation. The higher the temperature, the faster the rate of electron hole pair generation and migration, i.e., the larger the dark current, the more it increases sensor noise and directly affects the signal-to-noise ratio [32]. At lower temperatures, the activation energy of ether molecules is insufficient, making it difficult to adsorb onto the sensing material, resulting in a low response. As the temperature increases, ether molecules gain more activation energy to overcome energy barriers and react with adsorbed oxygen molecules. When the temperature exceeds 153 °C, the signal strength of the sensor also decreases. Excessive temperature leads to an increase in the desorption rate of ether molecules on the sensing material [26,33], resulting in a decrease in reaction performance. On the other hand, thermodynamic laws indicate that under high temperatures, the great average kinetic energy of electrons, and the high uncertainty in the conversion, transfer and output processes of photocharges, leading to an increase in instrument noise. The signal-to-noise ratio of the sensor decreases sharply with the increase in temperature. Based on the signal-to-noise ratio, 153 °C was selected as the most suitable operating temperature for the next step of research.

### 2.3. Effect of Flow Rate on CTL Intensity

**Figure 5:**
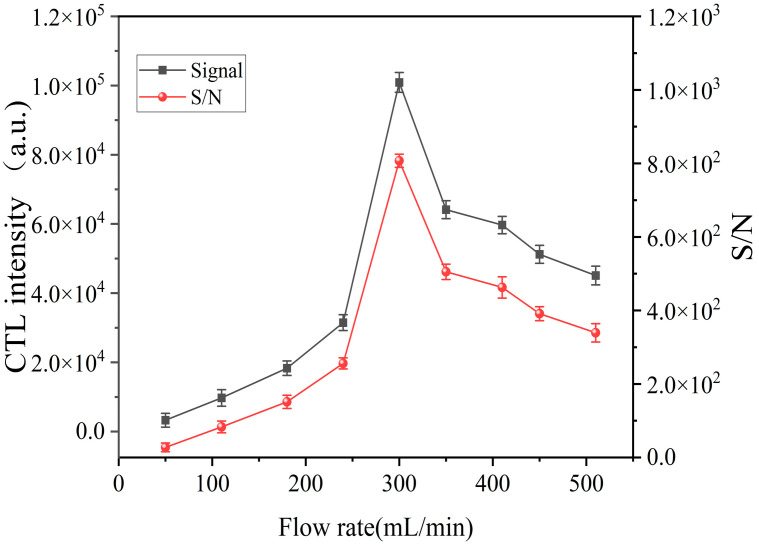
Effects of flow rate on CTL intensity and S/N (concentration: 25 ppm; operating temperature: 153 °C).

As a major factor, the carrier gas flow rate has a considerable influence on the performance of CTL-sensitive materials. The CTL intensity and the corresponding S/N are plotted in Figure 5 for different carrier gas flow rates (range: 50–510 mL/min.) It can be observed that in the range of 50–300 mL/min, the CTL intensity and S/N increase with the increase in the carrier gas flow rate, and both reach their peak at the same time at the carrier gas flow rate of 300 mL/min. It is indicated when the gas flow rate is relatively low that the catalytic reaction rate oxidation process mainly depends on the transfer rate of the sample gas. When the flow rate increases, it promotes the replacement rate of the gas around the sensor, thus promoting the reaction between the SnS and oxygen and shortening the recovery and response times [34]. At the same time, the gas flow takes away carbon dioxide and water vapor, thus promoting the reaction. However, this effect reaches saturation as the gas flow rate increases. As the gas flow rate increases, the amount of oxygen molecules adsorbed on the material surface becomes high. However, at the critical value, the yield of CTL intermediates is proportional to the chemisorbed oxygen content, and an excessive chemisorbed oxygen content will inhibit the yield of CTL intermediates, thus affecting the CTL [35]. When the carrier gas flow rate is too low, the effective contact concentration between the catalyst and the gas is low, resulting in a lesser signal intensity. As the flow rate continuously increases, the amount of ether gas in contact with the sensor surface per unit of time increases, and the CTL intensity tends to rise. When the flow rate exceeds 300 mL/min, part of the ether gas is taken out of the reaction chamber before reacting with the catalyst, resulting in insufficient contact time and the reaction of gas molecules on the surface of the composite catalyst. On the other hand, the excess chemisorbed oxygen inhibits the CTL [17]. Therefore, 300 mL/min was chosen as the optimal air flow rate.

### 2.4. Effect of Concentration

**Figure 6:**
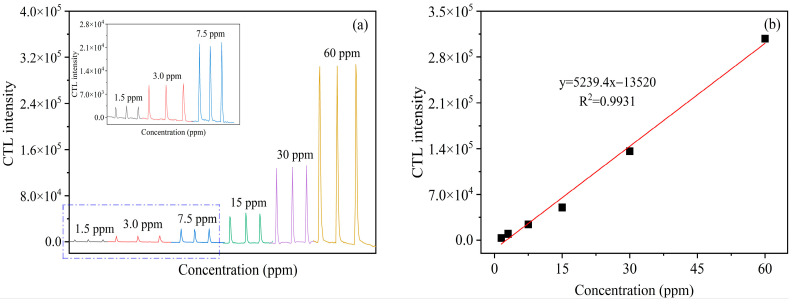
(**a**) Response of CTL sensor to different ether concentrations (black line: 1.5ppm; red line: 3.0 ppm; blue line: 7.5 ppm; green line: 15 ppm; purple line: 30 ppm; yellow line: 60 ppm); (**b**) the calibration curve of CTL intensity (flow rate: 300 mL/min; operating temperature: 153 °C).

The analytical characteristics of CTL for ether under optimal conditions were further investigated. As shown in Figure 6a, the CTL sensing performance of the nano-SnS material was evaluated according to the response of the CTL system to 1.5 ppm, 3.0 ppm, 7.5 ppm, 15 ppm, 30 ppm, and 60 ppm of ether. Figure 6a shows the CTL signal curves corresponding to a range of concentrations of ether vapor, and it can be seen that the corresponding CTL signal intensity increases with increasing concentration. Figure 6b shows the calibration curve of CTL intensity versus ether concentration, and the results show that SnS has a sensitive and stable CTL response to ether. Moreover, the CTL intensity increases with increasing ether concentration. In the range of 1.5–60 ppm, the CTL intensity shows a good linear relationship with the concentration of ether, and the linear regression equation is y = 5239.4x − 13,520 (R^2^ = 0.9931), where y is the average intensity of CTL produced by ether on the surface of nanomaterials after six parallel experiments, x is the concentration of ether, and R is the correlation coefficient. The detection limit was calculated according to the following equation [36], yielding a detection limit of 0.15 ppm (S/N = 3, where S denotes signal and N denotes noise). Compared to some of the other materials in Table 2, the SnS sensor displays a low detection limit, enabling the possibility of detecting ether at low concentrations.

(1) $$D=3N\times \frac{Q}{I}$$

In relation to the detection range, Q signifies the lowest measurable concentration of intake air, and N refers to the noise level corresponding to this minimal intake concentration detectable within the established range. Additionally, I represents the signal response value associated with the least air intake concentration detectable within the detection range. 

### 2.5. Selectivity and Stability of the Sensor

**Figure 7:**
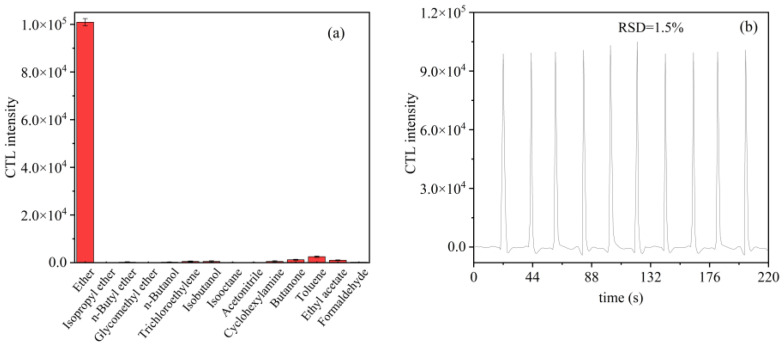
(**a**) CTL response of the sensor to different compounds; (**b**) the results obtained from ten repetitions of SnS determination (flow rate: 300 mL/min; operating temperature: 153 °C; concentration: 25 ppm).

In order to investigate the selectivity of CTL sensors based on SnS nanomaterials, some possible interfering substances were used to investigate the selectivity for ether sensing under pre-optimized conditions. All experiments were carried out under the same conditions, and the same concentration of ether was chosen for the detection of other VOCs. The corresponding response signals were obtained after repeated measurements. We chose several representative VOCs such as ethers, alcohols, and ketones for the selectivity experiments. Thirteen VOCs were tested, including isopropyl ether, n-butyl ether, glycomethyl ether, n-butanol, trichloroethylene, isobutanol, isooctane, acetonitrile, cyclohexylamine, butanone, toluene, ethyl acetate, and formaldehyde. CTL intensity levels are shown in Figure 7a, which shows that ether has high selectivity compared to the other VOCs. It is observed that although other gases can also be catalyzed on the nanomaterials, ether generates a much larger number of CTL intermediates than others. It can therefore be concluded that the sensor has a good selectivity for ether.

In addition to excellent selectivity, stability is also essential for the sensor. After 6 weeks, the stability of the sensor was tested again by injecting the same concentration of ether vapor into the reaction chamber 10 times in a row under optimal conditions. The results of this are shown in Figure 7b. It can be seen that the change in CTL signal intensity for these 10 measurements is small and reproducible, and the relative standard deviation (RSD = 1.5%) is less than 3.0%, indicating that the CTL sensor detects ether with less fluctuation and good repeatability.

### 2.6. Advantages of SnS Sensors

In Table 2, compared to some CTL sensors based on ZnWO_4_, SiO_2_/Fe_3_O_4_, Mg-Al-layered double oxide (Mg-Al LDO), CdO, Al-Fe composite oxide, and TiO_2_, our sensor shows the advantages of a low operating temperature and short recovery time. Its low operating temperature provides the possibility of practical use. In addition, a high sensitivity is necessary for a sensor, which makes the sensor presented here one with great potential for practical use.

**Table 2:** Comparison of CTL methods for detecting ether using different materials.

Test Samples	Sensing Material	Response Time (s)	Recovery Time (s)	Operating Temperature (°C)	Linear Range (ppm)	LOD (ppm)	References
diethyl ether	ZnWO_4_	3	7	330	20–3500	8.7	[15]
	SiO_2_/Fe_3_O_4_	5	30	320	10–3000	6.7	[16]
	Mg-Al LDO	2.5	15	205	7–593	1.5	[17]
	α-MoO_3_	16	2	120	9–2000	7.5	[37]
	CdO	3	10	285	10–4000	6.5	[38]
	Al-Fe composite oxide	4	8	180	10–5800	4.3	[39]
	TiO_2_	—	—	200	148–3706	111.2	[40]
	SnS	3	8	153	1.5–60	0.15	This work

### 2.7. Mechanistic Discussion

The gas-sensing mechanism of SnS can be explained with the surface control model. SnS is a common n-type semiconductor [19]. When SnS is exposed to the air, oxygen molecules adsorb on the surface of SnS and capture electrons [41], and the physically adsorbed oxygen molecules will transform into chemically adsorbed oxygen molecules, forming the oxygen ion $O_{2}^{-}$, as shown in the following equation:

(2) $$O_{2}(\mathrm{gas})\to O_{2}(\mathrm{ads})$$

(3) $$O_{2}(\mathrm{ads})+e^{-}\to O_{2}^{-}$$

On the surface of SnS, an electron depletion layer (EDL) is formed due to the adsorption of oxygen. When the SnS is exposed to ether gas, oxygen molecules adsorbed on the surface react with the ether gas. This reaction causes the trapped electrons to be released back into the conduction band of the SnS [42]. This process results in a reduction in the thickness of the EDL. In addition, a luminescence phenomenon occurs when the excited-state intermediates return to the ground state. This phenomenon is ascribed to the release of energy in the form of photons when electrons transfer from higher to lower energy levels.

**Figure 8:**
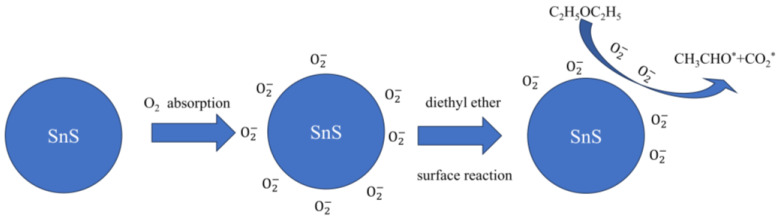
Possible mechanism of the CTL activity of diethyl ether on the surface of SnS nanomaterials (“*” for excited state intermediates).

According to the commonly accepted theory of CTL reaction [43], ether will form excited state intermediates upon catalytic oxidation on the surface of SnS nanomaterials. Since these excited state intermediates have a high energy, they will fall into the ground state quickly and release energy. Therefore, the luminescence phenomenon occurs when ether is catalytically oxidized on the surface of SnS. Some reports have suggested that ether forms excited state CH_3_CHO* and CO_2_* molecules during catalytic oxidation and speculate that the products of this are acetaldehyde and carbon dioxide [44,45]. The off-gas produced by catalytic oxidation of ether molecules on the surface of SnS nanomaterials is carbon dioxide [46]. Therefore, the mechanism of the CTL activity of ethyl ether on SnS nanomaterials is shown in Figure 8: (1) under certain conditions, O_2_ adsorbed on the surface of SnS captures electrons to generate $O_{2}^{-}$; (2) $O_{2}^{-}$ reacts with ethyl ether to generate intermediate CH_3_CHO* and CO_2_* molecules; and (3) the excited CH_3_CHO* and CO_2_* molecules return to the ground state and generate luminescent signals. The processes involved are as follows [47,48,49,50]:

(4) $$O_{2}+e^{-}\to O_{2}^{-}$$

(5) $$C_{2}H_{5}OC_{2}H_{5}+O_{2}^{-}\to CH_{3}CHO*+CO_{2}*+\mathrm{hv}$$

(6) $$CH_{3}CHO*\to CH_{3}CHO+\mathrm{hv}$$

(7) $$CO_{2}*\to CO_{2}+\mathrm{hv}$$

## 3. Materials and Methods

### 3.1. Test Reagents and Instruments

The chemical reagents used in this experimental process include: stannous chloride (SnCl_2_·2H_2_O, 98.0%, Shanghai Sinopharm Chemical Reagent Co., Ltd., Shanghai, China); anhydrous ethanol (C_2_H_6_O, 99.5%, Zhengzhou Yibang Industrial Co., Ltd., Zhengzhou, China); thiourea (CH_4_N_2_S, 99.0%, Shanghai Rinn Technology Development Co., Ltd., Shanghai, China); anhydrous ethylene glycol (C_2_H_6_O_2_, 99.99% Ltd., Shanghai, China); and ultrapure water (18.25 MΩ*cm, FST-TOP ultrapure water equipment, Shanghai Fushit Instrument Co., Shanghai, China).

The devices used in the experimental process include: a high-voltage stabilized power supply (NL01TP1100, Beijing Xinzhuo Bo Yu Optical and Mechanical Equipment Co., Ltd., Beijing, China); miniature vacuum pump (VM7002-5V, Chengdu Ruiyi Mechanical Design Centre, Chengdu, China); electric blow-drying oven (DHG-9023A, Shanghai Yiheng Science and Technology Co., Ltd., Shanghai, China); contact voltage regulator (TDGC2-2, Zhejiang Zhentai Electric Apparatus Co., Ltd., Zhejiang, China); and magnetic stirrer (RCT basic, Aika Instrument Co., Ltd., Staufen, Germany)

### 3.2. Preparation of Nanomaterials

The SnS nanomaterials were prepared using a hydrothermal method. A total of 0.54 g of SnCl_2_·2H_2_O was dissolved in 60 mL of anhydrous ethylene glycol, and the mixed solution was stirred magnetically for 10 min to achieve a uniform dispersion. Then, 0.36 g of thiourea was added to the above mixed solution and stirred for 30 min. The mixed liquid was then poured into a 100 mL Teflon-lined stainless steel reactor and heated to 180 °C for 12 h. After the reaction was completed, the sample was taken out and cooled to room temperature, washed with deionized water and ethanol alternately 6 times, and then dried in an oven at 70 °C for 8 h to obtain SnS powders.

### 3.3. Main Analytical Instruments

The main analytical instruments used in the experiments were as follows: the surface morphology of the prepared nanomaterials was analyzed using a scanning electron microscope (Zeiss, Jena, Germany); an EDS (energy-dispersive spectrometer, AURIGA, Jena, Germany) was used to analyze the chemical composition of the prepared materials; an X-ray diffractometer (Panalytical, Almelo, The Netherlands) was used to analyze the components and crystal structure of the materials; a Fourier infrared spectrometer (NEXUS-870, Thermo Fisher Corporation, Massachusetts, USA) was employed for analyzing functional groups on the surface of material samples; and measurements of gas luminescence sensing signals were obtained using the BPCL-1-TIC (BPCL Ultra-Weak Chemiluminescence Analyzer) (Guangzhou Microlight Technology Co., Ltd., Guangzhou, China).

### 3.4. CTL Device and Detection Method

**Figure 9:**
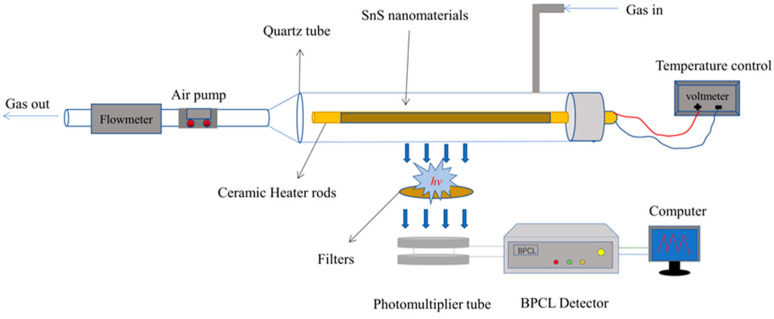
Schematic diagram of the BPCL-1 sensor device.

The system schematic diagram of the BPCL Ultra-Weak Chemiluminescence Analyzer (BPCL-1-TIC, Guangzhou Microlight Technology Co., Ltd., Guangzhou, China) is shown in Figure 9. Its system consists of three parts, as follows: (I) Reaction chamber: this contains a ceramic heating rod coated with a layer of sensitive material and a quartz tube (with gas inlet and outlet), which are placed together in a square light-proof chamber. The gas sample is injected via a syringe, and passed into the quartz tube with the carrier gas in full contact with the material on the surface of the ceramic heating rod; (II) Temperature and flow rate control device: this comprises a temperature control system for regulating the temperature of the ceramic heating rod and flow rate control system, in turn regulating the flow rate of the carrier gas through the air pump; (III) Photoelectric detection and data processing system: this consists of filters, photomultiplier tubes, and BPCL analyzers. The core detector device in the BPCL-1 is a photomultiplier tube (PMT), which is detected via a single photon and transmitted to an amplifier and amplified with a high voltage current. The amplifier converts the analog current into a digital current, which transmits the light-emitting signal to a computer and calculates it to produce the result. When starting the measurement, the instrument maintains the counting stability, and we can automatically measure the background noise value.

The concentration of the target gas is calculated from the saturated vapor pressure equation [51]:

(8) $$C=\frac{V_{i}\times P_{0}}{V_{c}\times P_{a}}$$

where C is the target concentration of VOC, V_i_ is the volume of VOC inhaled into the syringe, V_c_ is the volume of the laboratory, P_0_ is the vapor pressure of the gas to be tested at room temperature, and Pa is the standard atmospheric pressure.

## 4. Conclusions

In this work, SnS nanomaterials were synthesized using a simple hydrothermal method, and their cataluminescence properties towards ether were investigated. The optimal reaction conditions were determined to be 153 °C and 300 mL/min. Under the optimized conditions, the CTL intensity was proportional to the concentration of ether in the range of 1.5–60 ppm, with a linear regression equation of y = 5239.4x − 13520 (R^2^ = 0.9931, n = 6) and a low detection limit of 0.15 ppm. The selectivity and stability of the sensor were also investigated. The experiment shows that this sensor has a high selectivity to ether; the relative standard deviation of 10 measurements in the stability test (RSD = 1.5%) was much less than 3.0%, indicating that the sensor has a good stability. Compared with some other sensing materials, SnS nanomaterials not only have low operating temperatures, but also have a high sensitivity, high selectivity, and good stability for the detection of ether. It is expected that SnS nanomaterials provide a new idea for the fabrication of highly selective ether gas sensors with low operating temperatures.

## Figures and Tables

**Table 1:** The elemental content of the SnS.

Elements	Atomic%
S	52.87
Sn	47.13
Total	100.00

## Data Availability

Data are contained within the article.
